# A time-course microarray data analysis reveals consistent dysregulated genes and upstream microRNAs in autoantibody-mediated arthritis

**DOI:** 10.1186/s13018-017-0674-0

**Published:** 2017-12-02

**Authors:** Xinwen Wang, Jie Bai, Zhen Jia, Yangjun Zhu, Jijun Liu, Kun Zhang, Dingjun Hao, Lisong Heng

**Affiliations:** 1 Department of Orthopedics, Honghui Hospital, Xi’an Jiaotong University, 555 East Youyi Road, Xi’an, 710054 Shaanxi People’s Republic of China; 2 Department of Endocrinology, Xi’an No. 1 Hospital, Xi’an, 710002 Shaanxi People’s Republic of China

**Keywords:** Arthritis, Differentially expressed genes, MicroRNA, Regulatory network

## Abstract

**Background:**

The purpose of this study is to identify key genes and microRNAs (miRNAs) involved in autoantibody-mediated arthritis (AMA).

**Methods:**

A time-course microarray data (ID: GSE27492) of peripheral blood leukocytes, ankle tissue, and synovial fluid from K/BxN mouse serum-transferred mice were downloaded from Gene Expression Omnibus. Those samples were collected at days 0, 1, 3, 7, 12, and 18 after serum injection. Limma of *R* was employed to identify differentially expressed genes (DEGs) in samples collected at days 1–18 compared with those collected at day 0. Consistent DEGs were obtained by taking the interaction of DEGs from different time points, followed by functional enrichment analysis. MiRNAs were screened out and constructed into regulatory network with DEGs using Cytoscape.

**Results:**

In total, 17 consistent DEGs were obtained, including downregulated *Ephx1* and upregulated *AF251705*, *Adam8*, *Arg1*, *Basp1*, *Ccl2*, *Ccl7*, *Ccl9*, *Ccr2*, *Clec4a2*, *Clec4d*, *Cxcl1*, *Fabp5*, *Fcgr1*, *Gp49a*, *Il1rn*, and *Saa3*. Those DEGs were associated with biological processes of immune response, inflammatory response, and defense response; chemokine signaling pathway; cytokine-cytokine receptor interaction; and NOD-like receptor signaling pathway. Additionally, 202 miRNAs were identified to have a regulatory effect on 9 of the 17 DEGs. Notably, miR-944, miR-374a, and miR374b were found to regulate the expression of *Cxcl1*, *Ccl7*, and *Ccl2*. *Clec4d* was targeted by 78 miRNAs.

**Conclusions:**

Our study reveals that 17 DEGs and 202 miRNAs may be associated with autoimmune disorder in the progression of AMA, which could guide future researches.

## Background

Rheumatoid arthritis (RA) is an autoimmune disease, which is characterized by inflammation of the joints and surrounding tissues and destruction of bone and cartilage [1]. Inflammatory response and immune response are found to play critical roles in the pathogenesis of RA [2–4]. Many cytokines have been implicated in the pathogenesis of arthritis, such as interleukin-1 (*IL-1*) [5], *IL-6* [6], *IL-18* [7], and CC motif chemokine ligand 13 [8]. Matrix metalloproteinases (MMPs) play key roles in the destructive process and thus are also implicated in arthritis [9], especially *MMP-1* and *MMP-13*. Those uncovered genes demonstrate an involvement of the gene factor in the progression of arthritis.

Mouse models with autoantibody-mediated arthritis (AMA) are widely used in researches of arthritis [10]. The K/BxN mouse model of arthritis is a common AMA mouse model, in which the upstream adaptive immune response involved in generation of arthritogenic autoantibodies can be easily separated from the downstream autoantibody-mediated effector phase [10]. Using this model, previous studies have demonstrated the involvement of immune response in the incidence of AMA. It is reported that mast cells contribute to the initiation of AMA via *IL-1* [11]. Monach et al. indicated that circulating C3 was necessary and sufficient for induction of AMA [12]. Neutrophils also take parts in the development of AMA [13]. Follicular dendritic cells (FDCs) are important for the induction of protective T cell-dependent humoral responses, and thus, they play critical roles in the initiation of AMA [14]. Thus, the K/BxN mouse serum transfer model could effectively help to reveal the underlying molecular mechanisms involved in the progression of AMA.

MicroRNAs (miRNAs) representing a family of non-coding RNAs are shown to play an essential regulatory effect on the innate immune system in rheumatic diseases [15]. MiRNA-30a-3p has a major regulatory role in regulating the autoimmune responses in arthritis by functioning as a basal repressor of tumor necrosis factor (ligand) superfamily, member 13b [16]. Inhibition of miR-155 can reversely increase the release of Src homology 2 domain-containing inositol 5-phosphatase 1 and thus suppress inflammatory response in arthritis patients [17]. MiRNA-22 exerts suppressive effect on cysteine-rich protein 61 expression which plays vital roles in mediating the joint inflammation and damage in arthritis [18]. Targeting the miRNAs associated with the progression of arthritis may serve as promising therapy strategies.

To systemically investigate the molecular mechanisms underlying AMA, the time-course gene expression profiles deposited by a previous study [10] were re-analyzed with bioinformatic tools. Consistent differentially expressed genes (DEGs) during the development of AMA in the K/BxN mouse serum transfer model were identified, followed by enrichment analysis of DEGs in an attempt to explore the molecular mechanisms. MiRNA-targeting DEGs were also predicted in order to provide an insight into the regulatory network of DEGs.

## Methods

### A time-course microarray data

Microarray data set GSE27492 deposited by Jacobs et al. [10] was downloaded from Gene Expression Omnibus, including 17 peripheral blood leukocytes samples, 19 ankle tissue samples, and 13 synovial fluid samples. Synovial fluid cells were collected from articular cavity of dissected ankles, and ankle tissue cells were from the dissected ankle joints. All the samples were collected from a mouse model of AMA at six time points after arthritis induction: day 0 (baseline), day 1 (preclinical), day 3 (disease onset), day 7 (early disease), day 12 (disease plateau), and day 18 (late disease). Notably, an autoimmune response can develop in K/BxN mice with the production of pathogenic anti-GPI antibodies which deposit on joint surfaces and induce arthritis [19]. Thus, AMA was induced in chemokine and chemokine receptor knockout mice on B6 background by intraperitoneal injection of 200 μL of K/BxN serum from 8-week-old K/BxN mouse, and the mouse model of AMA was established. Affymetrix Murine Genome U74A Version 2 Array was used to collect the gene expression profiles of RNA extracted from the three groups of samples.

### Screening of differentially expressed genes

Raw data were processed through background correction and median normalization using robust multi-array average method [20] from package Bioconductor of *R*. For the several probes corresponding to a single gene, their expression levels were averaged as the final expression level for the gene. Differential expression analysis was performed with package limma [21]. |log FC (fold change)| > 1 and *P* value < 0.05 were set as the cut-offs to screen out DEGs. The expression levels of genes from samples collected at day 1, 3, 7, 12, and 18 were separately compared with those from samples collected at day 0. The consistent DEGs were obtained for further analysis by taking the intersection of the DEGs emerging at all of the five time points.

### Functional enrichment analysis of the differentially expressed genes

To identify altered biological pathways in arthritis, Gene Ontology (GO) enrichment analysis [22] and Kyoto Encyclopedia of Genes and Genomes (KEGG) pathway analysis [23] were carried out for the DEGs using hypergeometric test provided by DAVID (Database for Annotation, Visualization and Integration Discovery, http://david.abcc.ncifcrf.gov/) [24]. *P* value < 0.05 was chosen as the threshold to screen out significant GO terms and KEGG pathways.

### Construction of regulatory network between microRNAs and differentially expressed genes

The miRNA-targeting DEGs were predicted based on 10 databases covering DIANA, MiRanda, MiRDB, MiRwalk, RNAhybrid, TargetScan, RNA22, PITA, PICTAR5, and PICTAR4. The predicted miRNAs from more than 5 of the 10 databases were identified as candidate miRNAs. Following, the regulatory network between miRNAs and DEGs was constructed using Cytoscape software [25].

## Results

### Identification of DEGs

**Fig. 1:**
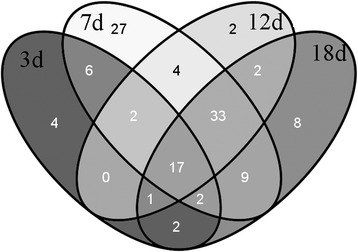
The Venn diagram of the differentially expressed genes identified at various time points (days 3, 7, 12, and 18). Totally, 17 differentially expressed genes consistently appeared at each time point

Compared with the samples collected at day 0 after K/BxN serum injection, a total of 0, 35, 103, 62, and75 DEGs were separately obtained from the samples collected at day 1, 3, 7, 12, and 18. The Venn diagram of the DEGs from different time points was shown in Fig. 1. By taking the intersection of the DEGs from different time points, a total of 17 consistent DEGs were obtained, including downregulated *Ephx1* (epoxide hydrolase 1, microsomal) and upregulated *AF251705* (cDNA sequence AF251705), *Adam8* (a disintegrin and metallopeptidase domain 8), *Arg1* (arginase, liver), *Basp1* (brain abundant, membrane attached signal protein 1), *Ccl2* (chemokine (C-C motif) ligand 2), *Ccl7* (chemokine (C-C motif) ligand 7), *Ccl9* (chemokine (C-C motif) ligand 9), *Ccr2* (chemokine (C-C motif) receptor 2), *Clec4a2* (C-type lectin domain family 4, member a2), *Clec4d* (C-type lectin domain family 4, member d), *Cxcl1* (chemokine (C-X-C motif) ligand 1), *Fabp5* (fatty acid binding protein 5, epidermal), *Fcgr1* (Fc receptor, IgG, high affinity), *Gp49a* (glycoprotein 49 A), *Il1rn* (interleukin 1 receptor antagonist), and *Saa3* (serum amyloid A 3) (Table 1).

**Table 1:** The FC magnitude of 17 consistent DEGs

Gene	Log_2_FC_3d	Log_2_FC_7d	Log_2_FC_12d	Log_2_FC_18d
*AF251705*	1.305238	1.457843	1.423637	1.534935
*Adam8*	1.397521	1.545198	1.64087	1.397731
*Arg1*	3.216408	3.470759	3.253301	3.500893
*Basp1*	1.183961	1.402966	1.330427	1.417313
*Ccl2*	3.136399	2.754264	2.608425	2.605791
*Ccl7*	2.379615	1.747449	1.676018	1.364921
*Ccl9*	1.592837	1.25133	1.348077	1.222959
*Ccr2*	1.395741	1.265384	1.07037	1.021065
*Clec4a2*	1.344762	1.371277	1.486593	1.286852
*Clec4d*	2.328534	3.102078	3.12611	3.014611
*Cxcl1*	1.152963	2.378911	2.050006	2.15751
*Ephx1*	− 1.12827	− 1.5318	− 1.36148	− 1.05361
*Fabp5*	1.300109	1.492187	1.360936	1.174031
*Fcgr1*	1.565833	1.337319	1.266228	1.255231
*Gp49a*	1.08629	1.48513	1.472467	1.684876
*Il1rn*	2.356432	2.933974	2.905693	3.029609
*Saa3*	2.536273	3.692907	3.451593	4.212274

*FC* fold change; *DEGs* differentially expressed genes. Gene names: *AF251705* cDNA sequence AF251705; *Adam8* a disintegrin and metallopeptidase domain 8; *Arg1* arginase, liver; *Basp1* brain abundant, membrane attached signal protein 1; *Ccl2* chemokine (C-C motif) ligand 2; *Ccl7* chemokine (C-C motif) ligand 7; *Ccl9* chemokine (C-C motif) ligand 9; *Ccr2* chemokine (C-C motif) receptor 2; *Clec4a2* C-type lectin domain family 4, member a2; *Clec4d* C-type lectin domain family 4, member d; *Cxcl1* chemokine (C-X-C motif) ligand 1; *Ephx1* epoxide hydrolase 1, microsomal; *Fabp5* fatty acid binding protein 5, epidermal; *Fcgr1* Fc receptor, IgG, high affinity; *Gp49a* glycoprotein 49 A; *Il1rn* interleukin 1 receptor antagonist; *Saa3* serum amyloid A 3

### Functional enrichment analysis results

With the cut-off of *P* value < 0.05, totally 14 significantly enriched GO terms and 3 KEGG pathways were obtained (Table 2). GO enrichment analysis revealed the DEGs were related to biological processes of immune response, inflammatory response, and defense response (e.g., *Cxcl1*, *Ccl2*, *Ccr2*, *Fcgr1*, and *Ccl7*), molecular functions of chemokine activity, chemokine receptor binding, and cytokine activity (e.g., *Cxcl1*, *Ccl2*, *Ccl9*, and *Ccl7*), as well as cellular components of extracellular space and extracellular region part (e.g., *Cxcl1*, *Ccl2*, *Ccl9*, and *Ccl7*). KEGG pathway enrichment analysis uncovered the DEGs were associated with chemokine signaling pathway (*Cxcl1*, *Ccl2*, *Ccr2*, *Ccl9*, and *Ccl7*), cytokine-cytokine receptor interaction (*Cxcl1*, *Ccl2*, *Ccr2*, *Ccl9*, and *Ccl7*), and NOD-like receptor signaling pathway (*Cxcl1*, *Ccl2*, and *Ccl7*).

**Table 2:** The significantly enriched GO and KEGG terms of the 17 DEGs

Term	Number	*P* value	Gene lists
GO term			
GO_BP: immune response	11	5.86E−12	*Cxcl1*, *Ccl2*, *Gp49a*, *Ccr2*, *Il1rn*, *Ccl9*, *Clec4a2*, *AF251705*, *Clec4d*, *Fcgr1*, *Ccl7*
GO_BP: response to wounding	7	1.09E−06	*Cxcl1*, *Arg1*, *Ccl2*, *Ccr2*, *Saa3*, *Fcgr1*, *Ccl7*
GO_BP: inflammatory response	6	3.12E−06	*Cxcl1*, *Ccl2*, *Ccr2*, *Saa3*, *Fcgr1*, *Ccl7*
GO_MF: chemokine activity	4	4.66E−06	*Cxcl1*, *Ccl2*, *Ccl9*, *Ccl7*
GO_MF: chemokine receptor binding	4	5.05E−06	*Cxcl1*, *Ccl2*, *Ccl9*, *Ccl7*
GO_CC: extracellular space	7	1.65E−05	*Cxcl1*, *Arg1*, *Ccl2*, *Il1rn*, *Saa3*, *Ccl9*, *Ccl7*
GO_BP: defense response	6	8.70E−05	*Cxcl1*, *Ccl2*, *Ccr2*, *Saa3*, *Fcgr1*, *Ccl7*
GO_CC: extracellular region part	7	1.70E−04	*Cxcl1*, *Arg1*, *Ccl2*, *Il1rn*, *Saa3*, *Ccl9*, *Ccl7*
GO_MF: cytokine activity	4	4.92E−04	*Cxcl1*, *Ccl2*, *Ccl9*, *Ccl7*
GO_BP: chemotaxis	3	6.25E−03	*Ccl2*, *Ccl9*, *Ccl7*
GO_BP: taxis	3	6.25E−03	*Ccl2*, *Ccl9*, *Ccl7*
GO_CC: extracellular region	7	9.88E−03	*Cxcl1*, *Arg1*, *Ccl2*, *Il1rn*, *Saa3*, *Ccl9*, *Ccl7*
GO_BP: locomotory behavior	3	2.78E−02	*Ccl2*, *Ccl9*, *Ccl7*
GO_MF: carbohydrate binding	3	3.20E−02	*Clec4a2*, *Clec4d*, *Ccl7*
KEGG pathway			
mmu04062:chemokine signaling pathway	5	6.21E−05	*Cxcl1*, *Ccl2*, *Ccr2*, *Ccl9*, *Ccl7*
mmu04060:cytokine-cytokine receptor interaction	5	1.95E−04	*Cxcl1*, *Ccl2*, *Ccr2*, *Ccl9*, *Ccl7*
mmu04621:NOD-like receptor signaling pathway	3	3.08E−03	*Cxcl1*, *Ccl2*, *Ccl7*

*GO* Gene Ontology, *KEGG* Kyoto Encyclopedia of Genes and Genomes; *DEGs* differentially expressed genes; *BP* biological process; *MF* molecular function; *CC* cellular component. Gene names: *AF251705* cDNA sequence AF251705; *Arg1* arginase, liver; *Ccl2* chemokine (C-C motif) ligand 2; *Ccl7* chemokine (C-C motif) ligand 7; *Ccl9* chemokine (C-C motif) ligand 9; *Ccr2* chemokine (C-C motif) receptor 2; *Clec4a2* C-type lectin domain family 4, member a2; *Clec4d* C-type lectin domain family 4, member d; *Cxcl1* chemokine (C-X-C motif) ligand 1; *Fcgr1* Fc receptor, IgG, high affinity; *Gp49a* glycoprotein 49 A; *Il1rn* interleukin 1 receptor antagonist; *Saa3* serum amyloid A 3

### Regulatory network between microRNAs and differentially expressed genes

**Fig. 2:**
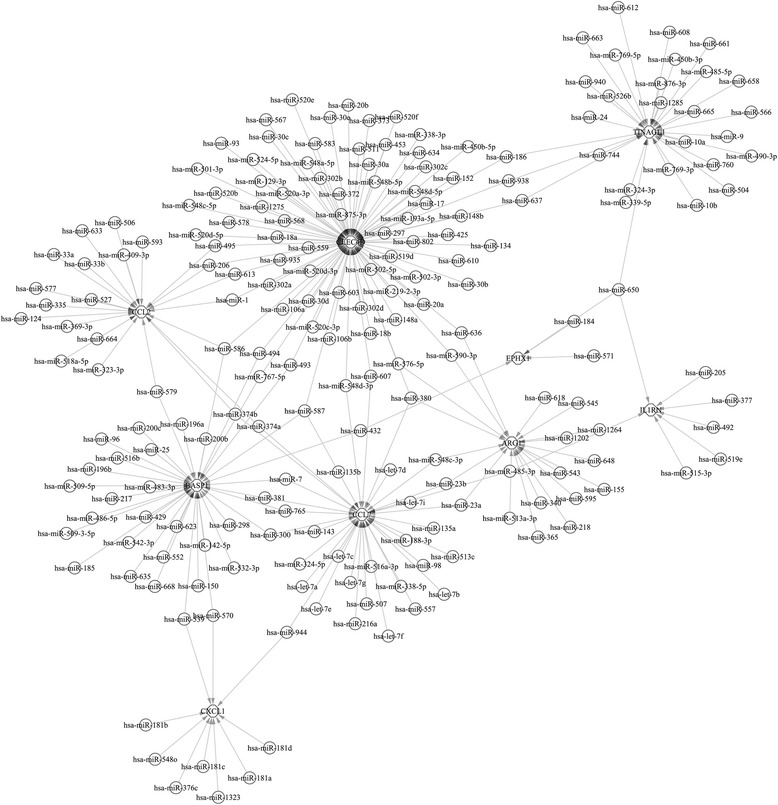
The regulatory network between 202 microRNAs and 9 differentially expressed genes. Circle represents microRNAs, and hexagon represents differentially expressed genes

Using the 10 databases for predicting miRNAs, 202 persuasive miRNAs were identified to have a regulatory effect on 9 of the 17 DEGs, including miR-155, miR-152, miR-124, miR-1, and miR-206 (Fig. 2). Importantly, according to the network, miR-944 was found to have a regulatory effect on the expression of *Cxcl1* and *Ccl7*; miR-374a and miR374b both could regulate the expression of *Ccl7* and *Ccl2*. Additionally, *Clec4d* was targeted by 78 miRNAs, suggesting an important role in the regulatory network.

## Discussion

Focusing on screening molecular targets for AMA therapy, this study obtained 16 up-regulated and 1 downregulated genes in the mouse with AMA compared with the mouse without AMA by re-analyzing the time-course microarray data. Large troop of miRNAs was also screened to have a regulatory effect on 9 of the 17 DEGs based on the predictive databases. Enrichment analysis unmasked the potential molecular mechanisms by which the DEGs take part in the development of AMA.

Chemokines consisting of CXC, CC, C, and CX_3_C groups and their receptors are revealed to play key roles in leukocyte migration to inflamed synovium in the treatment of chronic inflammatory disorders of RA [26]. Accordingly, this study revealed a number of dysregulated chemokines and their receptors in the development of AMA, such as *Adam8*, *Saa3*, *Il1rn*, *Ccl2*, *Ccl7*, *Ccl9*, *Ccr2*, and *Cxcl1*. GO enrichment analysis revealed that immune response and inflammatory response were significantly enriched biological processes of DEGs, which were linked to RA and in line with previous studies [2–4, 27]. Those biological processes-associated genes, such as *Cxcl1*, *Ccl2*, *Ccr2*, *Fcgr1*, and *Ccl7* were also associated with molecular functions of chemokine activity, chemokine receptor binding, and cytokine activity. Thus, a disorder of chemokine-associated functions was also observed in this study. In addition, *A*dam8 is detected under several pathological conditions and may be a potential drug target for the treatment of inflammatory diseases [28]. IL-33-induced neutrophil migration in RA is found to be dependent on *Cxcl1*, which may be a target of anti-TNF therapy [29]. *CCR2* is regarded as a therapeutic target in RA treatment [30]. These findings suggest that some chemokines and receptors as therapeutic targets may have feasibility and application prospects in RA therapy. However, the *CCR2* blockade cannot sufficiently induce clinical improvement in RA patients [31].The anti-*CCL2* monoclonal antibody treatment also results in poor clinical improvement [32]. It could be inferred that the chronic inflammatory disorder may not only result from multiple dysregulated chemokines and receptors but also result from some other immune-associated genes; thus, targeting single molecule may not improve and cure the disease. Further studies are still needed to confirm the clinical feasibility and application prospects of these dysregulated chemokines and their receptors in RA therapy. Furthermore, as previously demonstrated, *Fcgr1* makes a contribution to arthritis pathology [33]; *Clec4a2* and *Clec4d* play essential roles in maintaining the homeostasis of immune system [34, 35]; *Arg1* activity is important for wound healing functions and immune response to viral infections [36]. Therefore, the uncovered 17 dysregulated genes may jointly contribute to the chronic inflammatory disorder and need an integrated regulation.

Correspondingly, KEGG pathway enrichment analysis revealed the DEGs were associated with chemokine signaling pathway, cytokine-cytokine receptor interaction, and NOD-like receptor signaling pathway. Those pathways are closely related to inflammatory and immune responses. The upregulation of chemokine receptors *Ccr1*, *Ccr2*, and *Ccr5* are associated with macrophage and endothelial cell infiltration in arthritis [37]. The cytokine-cytokine receptor interaction is also revealed to be implicated in the progression of RA [38]. The nucleotide-binding oligomerization domain (NOD)-like receptors are a class of specialized intracellular receptors implicated in the innate immune system [39]. The *NOD2* signaling could be used as a therapeutic target for the control of RA [40]. Mesothelial cells involved in the inflammatory responses could secret chemokines *Cxcl1* and *Ccl2* by *Nod1* stimulation [41]. Considering the possible role of these pathways in regulating inflammatory and immune response, these specific pathways may provide a new insight for therapeutics of other autoimmune disorders, such as periodental infection (bacterial), urinary tract infection (bacterial), and psoriatic arthropathy. More studies are still needed to confirm our speculation.

Furthermore, a large cohort of miRNAs was identified to be associated with the DEGs and the miRNAs were constructed into a regulatory network with DEGs. MiR-155, miR-152, and miR-124 have been shown to be implicated in RA [42–44]. MiR-1 and miR-206 are also associated with inflammation [45]. They were all contained in our regulatory network, suggesting the reliability of our network to some extent. Additionally and importantly, based on the network, miR-944, miR-374a, and miR374b were revealed to have a regulatory effect on the expression of *Cxcl1*, *Ccl2*, and *Ccl7*. MiR-944 located in the intron of *TP63* gene could promote cell proliferation, migration, and invasion in human cervical cancer [46]. MiR-374 experiences dysregulation in the inflammatory mice [47]. Although there are no direct links between those two miRNAs with immune systems, based on our results, miRNA-944 and miR-374 were predicted to be involved in the AMA via targeting *Cxcl1*, *Ccl2*, and *Ccl7*. Additionally, *Clec4d* was targeted by 78 miRNAs. Previous report has pointed out activated *Clec4d* can contribute to Mincel expression and thus enhance the immune system [35]. It could be therefore speculated that *Clec4d* may play a role in AMA as a downstream target of various miRNAs. Although the role of the above miRNAs and *Clec4d* in RA development has not been fully investigated, based on our results, we speculate that these molecules may serve as potential targets for RA treatment. All these speculations need further research. There were some limitations in our study. The comparative studies of other arthritis models were not included this study. Moreover, the results were obtained based on the bioinformatic methods and should be further confirmed by mechanistic experiments. Even so, this study reveals 17 DEGs associated with AMA, which may provide a theoretical basis for further studies.

## Conclusion

Focusing on screening of therapeutic targets for AMA, this study reveals that 17 dysregulated genes may jointly and consistently play roles in the progression of AMA by affecting the immune system functions and pathways. MiR-944, miR-374a, and miR374b may be associated with AMA development by targeting some chemokines. Also, *Clec4d* probably mediates the functions of a large number of miRNAs in the development of AMA. Further experiments, both epidemiological and mechanical, should be performed to confirm the results.

## Abbreviations

AMA: Autoantibody-mediated arthritis
DEGs: Differentially expressed genes
FDCs: Follicular dendritic cells
GO: Gene Ontology
KEGG: Kyoto Encyclopedia of Genes and Genomes
miRNAs: MicroRNAs
NOD: Nucleotide-binding oligomerization domain
RA: Rheumatoid arthritis
